# Vis/NIR Spectroscopy and Vis/NIR Hyperspectral Imaging for Non-Destructive Monitoring of Apricot Fruit Internal Quality with Machine Learning

**DOI:** 10.3390/foods14020196

**Published:** 2025-01-10

**Authors:** Tiziana Amoriello, Roberto Ciorba, Gaia Ruggiero, Francesca Masciola, Daniela Scutaru, Roberto Ciccoritti

**Affiliations:** 1 CREA—Research Centre for Food and Nutrition, Via Ardeatina 546, 00178 Rome, Italy; 2 CREA—Research Centre for Olive, Fruit and Citrus Crops, Via di Fioranello 52, 00134 Rome, Italy; roberto.ciorba@crea.gov.it (R.C.); ruggierogaia98@gmail.com (G.R.); francesca.masciola@crea.gov.it (F.M.); daniela.scutaru@crea.gov.it (D.S.)

**Keywords:** total soluble solids, titratable acidity, dry matter, Vis/NIR hyperspectral imaging, Vis/NIR spectrophotometer, artificial neural networks

## Abstract

The fruit supply chain requires simple, non-destructive, and fast tools for quality evaluation both in the field and during the post-harvest phase. In this study, a portable visible and near-infrared (Vis/NIR) spectrophotometer and a portable Vis/NIR hyperspectral imaging (HSI) device were tested to highlight genetic differences among apricot cultivars, and to develop multi-cultivar and multi-year models for the most important marketable attributes (total soluble solids, TSS; titratable acidity, TA; dry matter, DM). To do this, the fruits of seventeen cultivars from a single experimental orchard harvested at the commercial maturity stage were considered. Spectral data emphasized genetic similarities and differences among the cultivars, capturing changes in the pigment content and macro components of the apricot samples. In recent years, machine learning techniques, such as artificial neural networks (ANNs), have been successfully applied to more efficiently extract valuable information from spectral data and to accurately predict quality traits. In this study, prediction models were developed based on a multilayer perceptron artificial neural network (ANN-MLP) combined with the Levenberg–Marquardt learning algorithm. Regarding the Vis/NIR spectrophotometer dataset, good predictive performances were achieved for TSS (R^2^ = 0.855) and DM (R^2^ = 0.857), while the performance for TA was unsatisfactory (R^2^ = 0.681). In contrast, the optimal predictive ability was found for models of the HSI dataset (TSS: R^2^ = 0.904; DM: R^2^ = 0.918, TA: R^2^ = 0.811), as confirmed by external validation. Moreover, the ANN allowed us to identify the most predictive input spectral regions for each model. The results showed the potential of Vis/NIR spectroscopy as an alternative to traditional destructive methods to monitor the qualitative traits of apricot fruits, reducing the time and costs of analyses.

## 1. Introduction

Apricot (*Prunus armeniaca* L.) is one of the most widely cultivated fruit trees. According to the FAOSTAT data platform, the global cultivated area was more than 579 thousand ha and the whole production was 3.9 million tonnes in 2022, reflecting their economic and nutritional importance [1]. Italy is the world’s fourth-largest producer with an annual production of 230 thousand tonnes (5.9% of the global production), following Turkey (20.4%), Uzbekistan (11.5%), and Iran (7.8%).

Apricots are widely praised by consumers due to their organoleptic and nutritional characteristics, especially for their sweetness and juiciness [2,3]. The fruits can be intended for fresh consumption or for the processing industry for use in cakes, jams, juices, ice cream, candies, and jellies. Fruit quality at purchasing is evaluated by consumers through visual inspection methods and focuses on external parameters such as color, size, shape, and the absence of defects. However, internal quality, such as total soluble solids (TSS) content, titratable acidity (TA), and dry matter (DM), is important for both fresh consumption and industrial processing applications. The traditional methods used to assess these attributes are destructive and time-consuming, often leading to increased costs and product wastage. Therefore, in recent years, there was an increasing trend towards the development of non-destructive systems based on digital sensors and spectroscopic techniques, which exploit the interactions between electromagnetic radiation and matter. These instruments can be used by growers and producers to detect fruit internal quality traits, including for apricots [4,5,6,7,8]. Among all tools on the market, portable visible and near-infrared (Vis/NIR) spectrophotometers guarantee numerous advantages to the end-users: a quick and easy quality inspection; high repeatability; no damage to the fruit surface; a cost-effective and pollution-free approach; and the simple use of software for processing spectral data [9]. The hyperspectral imaging (HSI) technique is often used in food quality monitoring [10]. With respect to IR spectroscopy, HSI links to computer vision technology and spectroscopy, obtaining a so-called hyperspectral cube, in which two dimensions represent the spatial distribution of the sample and the third its spectral content [11]. In this way, a hyperspectral image can describe the physical features and chemical constituents of a sample [11,12,13]. However, the use of these devices requires the calibration and validation of predictive models through sophisticated data analysis techniques. The selection of an optimal data modeling method for predicting quality parameters is a fundamental step toward accurate monitoring based on Vis/NIR spectroscopy. Partial least-squares regression (PLS) is a method widely used when models are built using highly correlated spectral data. PLS models consider linear relationships among spectral data and quality traits. This could affect the models’ performance dealing with nonlinear relationships. In recent years, models involving artificial intelligence techniques, such as artificial neural networks (ANNs), are increasingly being used to predict fruit quality parameters due to the ability to model complex, often nonlinear and prior unknown relationships between input and output variables [11,14,15,16,17,18]. Usual regression methods require core information of the corresponding chemical system and knowledge of the presence of functional groups. Moreover, the ANN technique has no limitations on input variables and requires no knowledge on the relationship among the input variables. The ANN can also be used for unstable, noisy, imprecise, and incomplete data [17]. Finally, to build robust models that can be applied in apricot fruit monitoring and during the post-harvest stage, it is necessary to consider the quality characteristics of a large number of apricot cultivars and to use different years as input data.

Our study aimed to evaluate the performances of a portable visible and near-infrared (Vis/NIR, 310–1100 nm) spectrophotometer and a portable Vis/NIR hyperspectral imaging device (HSI, 400–1000 nm) in the prediction of the main apricot quality traits. Specifically, the objectives of this study were (i) to highlight similarities and differences among seventeen apricot cultivars from a single experimental orchard; (ii) to develop calibration multi-cultivar and multi-year models from Vis-NIR spectral data using ANN algorithms to estimate key internal quality attributes (total soluble solids content, TSS; titratable acidity, TA; and dry matter, DM); (iii) to evaluate the prediction accuracy and the generalizability of the developed models using external datasets; and (iv) to identify the most predictive input spectral bands for each model. To the best of our knowledge, no studies with such a large dataset, based on fruit from 17 cultivars and two years, and with a comparison of the predictive ability of models (developed using the machine learning technique) from the two devices have been published for apricots.

## 2. Materials and Methods

### 2.1. Plant Material and Experimental Design

Seventeen apricot cultivars, named Aurora, Bella d’Imola, Bora, Bulida, Cafona I, Krupna Skopjanka, Marietta Milady, Muscat, Nella, Ninfa, NJA42, Portici, Precoce Toscana, Rubista, S. Castrense, Tardiva di Bordaneil, and Thyrinthos, widely grown in Italy, were considered in this study. Geographical origin, ripening time and parental lines of genotypes analyzed, according to the varietal lists are reported in Table 1 [[19,20]; https://plantgest.imagelinenetwork.com (accessed on 10 November 2024)]. The apricots came from the Italian National Fruit Germplasm Collection of CREA—Research Centre for Olive, Fruit and Citrus Crops of Rome, Italy (latitude: 41°47′16.4″ N, longitude: 12°34′11.3″ E, altitude: 86 m a.s.l.). For each cultivar and each year, thirty fruits were harvested from different plants at the commercial maturity stage, for two crop years (2023 and 2024). All plants had the same planting age. In order to obtain samples of homogeneous ripeness for each cultivar and between cultivars, the maturity level was assessed using a portable Vis instrument, namely, a DA-meter (TR Turoni, Forlì, Italy), as described by Amoriello et al. [7]. This instrument can help to determine the optimum harvest maturity, strongly related to a variety’s characteristics that affect fruit internal quality [21,22]. After each harvest, apricot samples were transported to the laboratory. Six fruits without mechanical damage or visible defects for each year and for each cultivar were randomly selected from the starting material. All samples were cleaned with MilliQ water (Millipore, Bedford, MA, USA), blotted with a paper towel, and prepared for the following analyses. Then, each apricot was individually subjected to non-destructive and destructive measurements.

A second dataset with measurements of four additional apricot cultivars (Biotipo A, Ottavianese, Pellecchiella, and Perla) was used to assess the performance of the developed models and validate them.

### 2.2. Spectral Data Acquisition with a Portable Vis/NIR Spectrophotometer

Spectra were collected twice on the two opposite sides of the apricot samples using a handheld Vis/NIR spectrophotometer (F-750 Produce Quality Meter, Felix Instruments, Camas, WA, USA) [23]. The spectral range of the instrument was between 310 and 1100 nm, and the data resolution was 3 nm. Spectral data were acquired and converted to second derivative form, through AppBuilter v2.2.6™ software (Felix Instrument, 1554 NE 3rd Ave Camas, WA 98607, USA). For each apricot, the average spectrum for each side was recorded by averaging 2 scans per side. Therefore, 408 spectra, related to the 408 apricot data (204 sample × 2 replicates) were collected (17 cultivars × 2 years × 6 samples × 2 replicates). The two replicates are defined as follows: one replicate (one side) is the average value of two scans and the second replicate (the opposite side) is the average value of two scans.

### 2.3. Spectral Data Acquisition with a Portable HSI Device

**Figure 1:**
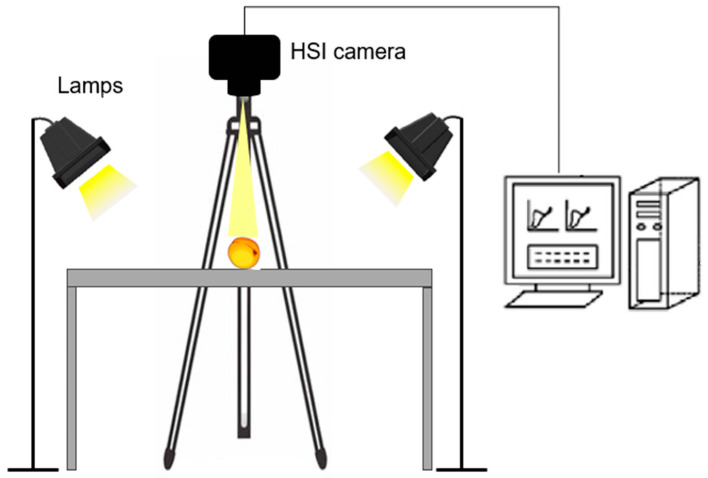
Schematic diagram of the Vis/NIR hyperspectral imaging system.

A portable Specim IQ hyperspectral camera with push-broom technology (Specim, Spectral Imaging Ltd., Oulu, Finland) was used to acquire hyperspectral images. The system (Figure 1) consisted of a spectrometer, a CMOS surface detector, a lens (Specimens FX10, Specim, Spectral Imaging Ltd., Oulu, Finland) with a detection range of 400–1000 nm and 224 wavelength points, and a computer with imaging acquisition software (Lumo-Scanner, Specim, Spectral Imaging Ltd., Oulu, Finland), as reported by Amoriello et al. [11]. The characteristics of the acquisition are as follows: a spatial resolution of 512 × 512 pixels, a spectral resolution of 7 nm resulting in 204 spectral bands across the wavelength range, and an integration time of 45.0 ms. A white diffuse reflectance target was used to achieve a white reference image to operate in simultaneous modality. The IQ studio software version 2019.05.29.2 (Specim, Spectral Imaging Ltd., Oulu, Finland) was used to gain the HSI images.

All HSI images were calibrated with white and dark reference images before processing, according to Amoriello et al. [11].

The images were processed using Evince software (version 2.7.12, Prediktera AB, Umeå, Sweden). A principal component analysis (PCA) algorithm was applied to portion out the images and to remove the background, as described by Amoriello et al. [24]. Then, the smoothing of the reflectance spectra was carried out with a baseline correction and the application of the first-order Savitzky–Golay filter for noise reduction. A standard normal variate (SNV) correction was used to reduce the light scattering. The mean spectrum was obtained as the average of the spectra related to all pixels of each sample, considering the overall HSI image.

### 2.4. Fruit Quality Traits

The skin color data were acquired on the external opposite sides of the fruit using a tristimulus colorimeter (Chroma Meter CR-200; Minolta, Milan, Italy) and expressed in the CIELab color space (L*, a*, b*) [25]. The sample dimensions (longitudinal diameter, lateral diameter, equatorial diameter), expressed in mm, were determined using a digital caliper (±0.05 mm accuracy). Fruit firmness (FF), expressed in Newton (N), was measured with a penetrometer (Fruit Pressure Tester FT011, TR snc, Forlì, Italy), using an 8 mm tip. The total soluble solids content (TSS), expressed in g 100 g^−1^ of FW, was determined with a digital refractometer (Refractometer 30PX, Mettler Toledo, Im Langacher, Greifensee Switzerland). Titratable acidity (TA), expressed in mEq L^−1^, was evaluated using an automatic titration system (785 DPM Titrino, Metrohm Ltd., Herisau, Switzerland). Dry matter (DM) was assessed by drying the fresh samples at 105 ± 1 °C until a constant weight was reached using an air-forced oven (Tecnocalor 2000, Tecnovetro srl, Monza, Italy), and the results were expressed as g 100 g^−1^ of fresh weight (FW). All determinations were performed in triplicate.

### 2.5. Chemometric Methods

#### 2.5.1. Data Analysis

In order to evaluate differences in all measured properties among the cultivars, and to compare the means of the measured traits among cultivars and identify statistically significant differences, the Tukey test was performed at a significance level of 5% using SPSS statistical software (version 22, SPSS, Chicago, IL, USA).

#### 2.5.2. Artificial Neural Network Models

**Figure 2:**
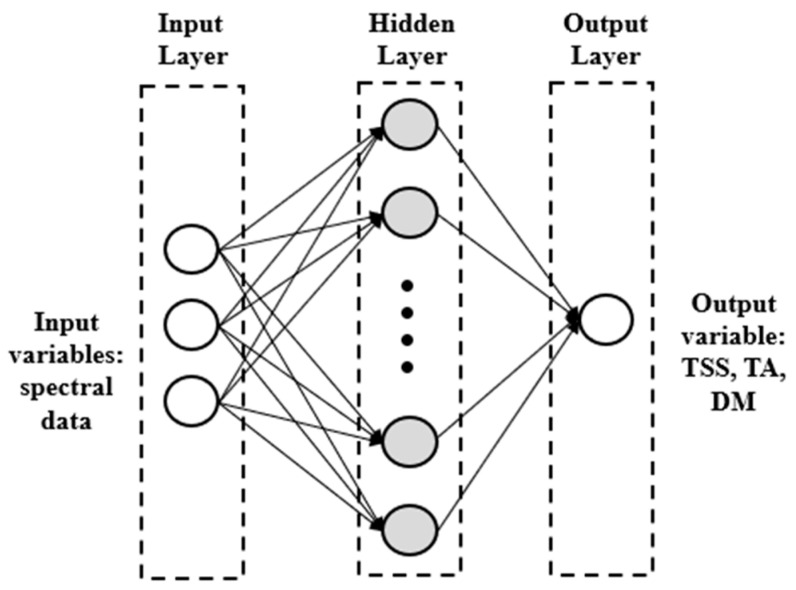
ANN-MLP structure.

A multilayer perceptron artificial neural network (ANN-MLP) combined with the Levenberg–Marquardt learning algorithm was employed to build models for predictions of three quality traits (TSS, TA, and DM). The neural network includes three main layers: an input layer; an output layer, i.e., the three quality attributes; and a hidden layer, as shown in Figure 2. The input layer contained 260 spectral data for the Vis/NIR dataset (from 320 to 1100 nm each 3 nm) and 202 spectral data for the HSI dataset (from 398 to 1004 nm each 3 nm).

In this study, the identity function, logistic sigmoid function, hyperbolic tangent function, and exponential function, as described by Amoriello et al. [25], were the activation functions applied in the hidden or output layers. Different topologies with different neurons in the hidden layer (from 1 to 50) were tested, and the training process of the network was run 100,000 times with random initial values of weights and biases. The best topology for the three quality parameters (TSS, TA, DM) was evaluated using prediction performance metrics: the coefficient of correlation between the observed and predicted values (r), the coefficient of determination (R^2^), the mean absolute error (MAE), and the root mean squared error (RMSE), as reported by Amoriello et al. [25].

The entirety of the two datasets, with data from all cultivars over the two years, was randomly split into three sub-datasets: 70% of the data (training set) for training, 15% (validation set) for validation, and the remaining 15% (test set) for testing of the models. Therefore, we used 288 data for the training set, 60 data for the test set, and 60 data for the validation set.

To identify the contribution of each input variable to the model, sensitivity analysis, based on the partial derivatives method and calculated for each variable and observation, was used. A global sensitivity is determined as the sum of the derivatives of the output of the k-th neuron in the output layer regarding the i-th input variable divided by the number of samples. The more important the variable is in building the model, the greater the global sensitivity is than 1.

To evaluate the models’ performances and their generalizability, two external datasets, one for the HIS device and one for the Vis/NIR spectrophotometer, were considered. The best topologies for each model and for the two devices were applied to the two external validation datasets. The external datasets consisted of 48 quality and spectral data from four different cultivars, namely, Biotipo A, Ottavianese, Pellecchiella, and Perla, collected during the harvests of 2023 and 2024. Specifically, 6 apricot samples × 2 replicates for each of the 4 cultivars were considered.

The models and sensitivity analyses were conducted using TIBCO^®^ Statistica statistical package software (version 13.5, TIBCO software Inc., Palo Alto, CA, USA).

## 3. Results

### 3.1. General Aspects of Apricot Fruit

A summary of the descriptive statistics (mean ± standard deviation) of the pomological characteristics of 17 apricot genotypes is shown in Table 2. The results indicated a significant variation among cultivars. The early–medium season cultivar Bora showed the highest weight and dimensions (weight: 70 ± 11 g; longitudinal diameter: 50 ± 4 mm; lateral diameter: 48 ± 3 mm; equatorial diameter: 47 ± 3 mm), whereas the medium–late season cultivar Muscat exhibited the lowest (weight: 30 ± 4 g; longitudinal diameter: 38 ± 2 mm; lateral diameter: 36 ± 3 mm; equatorial diameter: 34 ± 3 mm). All 17 cultivar samples showed superior quality, falling into the “Extra” class (longitudinal diameter must be ≥35 mm), according to Commission Regulation (EC) No 851/2000, which outlines the marketing standard for apricots [26].

The mean values of CIELab coordinates (L*, a*, b*) for each cultivar were used to describe the general coloring appearance of apricots (Table 3). All cultivars showed differences in fruit skin. Note that the Rubista cultivar has significantly different color coordinates from the others, with lower L* and b* values and higher a* values. The distinctive characteristics of 17 genotypes were in agreement with Ruitz et al. [27].

The physical (FF) and chemical (TSS, TA, DM) traits of apricots distinguished by cultivars are shown in Table 4. Regarding FF, significant differences (*p* < 0.05) were observed among the genotypes. Bora showed, on average, a greater flesh firmness (47 ± 36 N), while the lowest mean value was recorded for Krupna Skopjanka (8 ± 4 N). Furthermore, a considerable variability in firmness was observed for the Bora e NJA42 samples, indicating a different degree of ripening of the fruits. Among the genotypes, Muscat exhibited the highest TSS content (19.6 ± 2.1 g 100 g^−1^ FW), and Thyrinthos the lowest (11.2 ± 2.1 g 100 g^−1^ FW). All cultivars (Ninfa, Nella, Bora, Thyrinthos and Tardiva di Bordaneil) had an average level greater than or equal to 11.2 g 100 g^−1^ FW, considered the threshold value for marketing standard for apricot fruit. High variability between cultivars was also observed for titratable acidity.

The highest TA content was found for Rubista (417 ± 43 mEq 100 L^−1^) and the lowest for Portici (130 ± 30 mEq 100 L^−1^). Finally, there were significant differences in DM between the cultivars, although not related to the fruit ripening time. The variety with the highest mean dry matter level was Muscat (19.3 ± 2.5 g 100 g^−1^ FW), while the lowest was observed in Ninfa (9.4 ± 1.9 g 100 g^−1^ FW).

### 3.2. Spectrum Analysis

**Figure 3:**
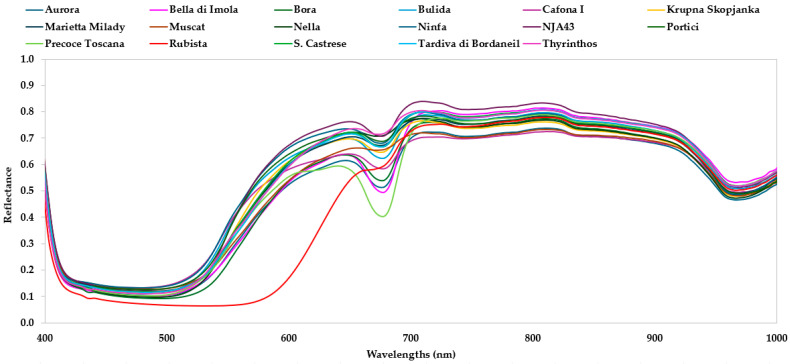
Mean reflectance spectra between 400 and 1000 nm wavelengths of 17 apricot genotypes, obtained with the HSI device.

**Figure 4:**
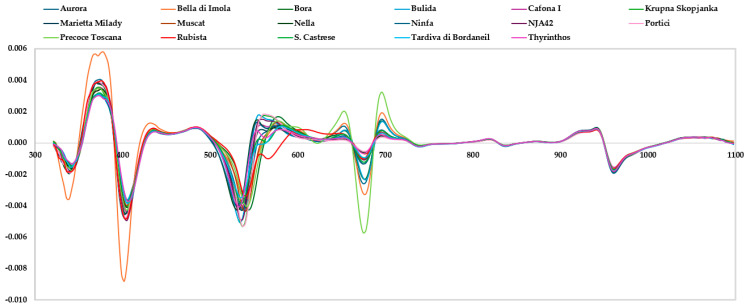
Mean second derivative absorbance spectra between 321 and 1100 nm wavelengths of 17 apricot genotypes, obtained with the Vis/NIR spectrophotometer. The *Y*-axis represents the second derivative absorbance with respect to wavelength.

The mean reflectance spectra acquired by the HSI device and the mean second derivative absorbance spectra acquired by the Vis/NIR spectrophotometer in the spectral regions (between 400 and 1100 nm for HSI and between 310 and 1100 nm for Vis/NIR spectrophotometer) of 17 apricot genotypes are shown in Figure 3 and Figure 4, respectively. In general, the spectral region between 310 and 1100 nm is described by overtone bands and multiple combinations of fundamental vibrations of -CH, -NH, and -OH groups related to the main structures of organic molecules [11,28,29]. The raw HSI spectra highlighted five main regions around 550–650 nm, 650–720 nm, 720–750 nm, 800–850 nm, and 920–1000 nm. Differently, the Vis/NIR spectrophotometer’s second derivative spectra showed 16 broad peaks around 340 nm, 370 nm, 400 nm, 432 mm, 490 mm, 540 nm, 550 nm, 570 nm, 630 nm, 680 nm, 700 nm, 821 nm, 840 mm, 920, 940 nm, and 960 nm. The spectral region between 340 and 500 nm is characterized by the visible absorption peaks of pigments, such as carotenes and xanthophylls [30]. In particular, β carotenoids in fruits have a color varying from yellow to orange-red, with a maximum absorption peak at 464 nm. Xanthophylls, lutein, and violaxanthins show absorbance peaks at approximately 435 nm and strong absorption between 350 and 500 nm [30]. Many authors associated the region between 530 and 580 nm to anthocyanins or to sugar–protein complexes [30,31,32]. The region at approximately 680 nm is related to the absorption of chlorophyll [7,30]. The peaks at around 760 nm and 970 can be assigned to the overtones of the OH groups related to water [4,33]. The spectral regions at approximately 830 nm, and between 950 and 1000 nm, are characteristic of the second overtone of the O–H and N–H groups, of the combination bands of the O–H bonds, and of the third overtone of the C– bonds [6,34].

### 3.3. ANN Models Based on Vis/NIR Spectrophotometer Data

**Figure 5:**
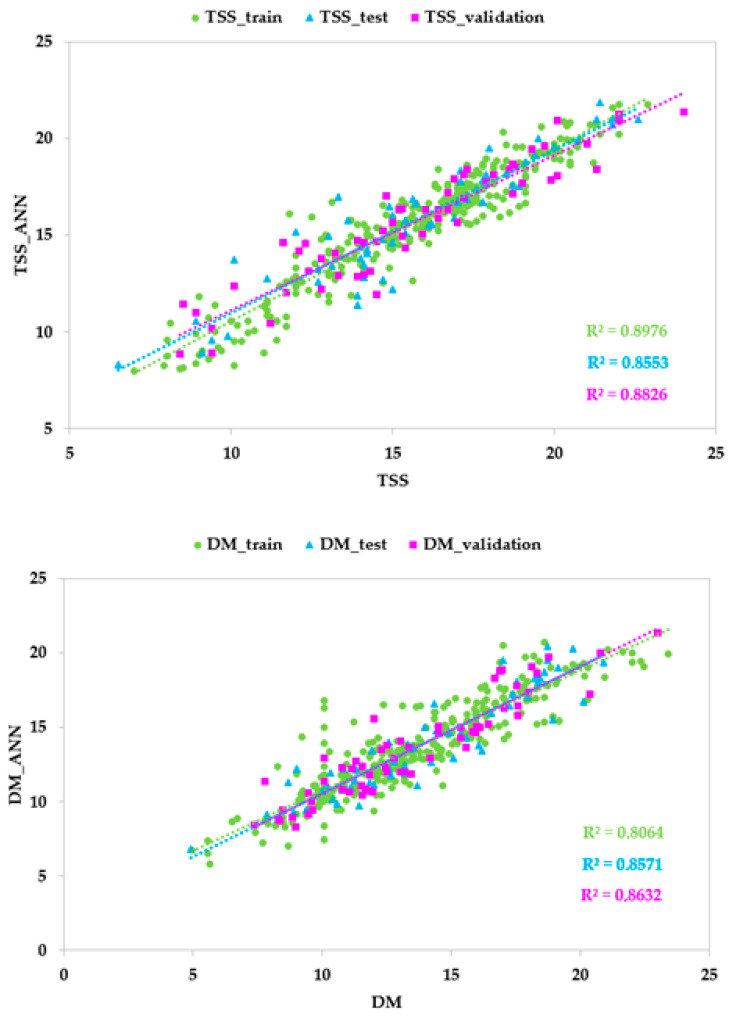

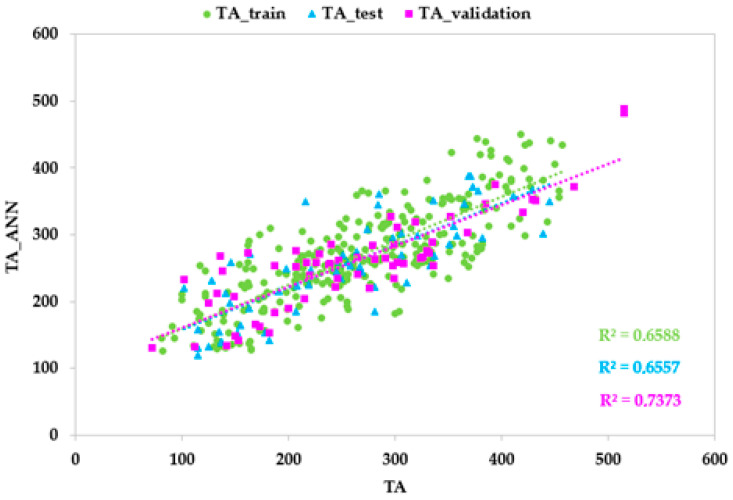
Predicted vs. experimental values of total soluble solids content (TSS), titratable acidity (TA), and dry matter (DM) using the optimal ANN topologies and Vis/NIR spectra. The coefficients of determination (R^2^) for training, test, and validation sets are reported.

The predictive performances of ANN models based on Vis/NIR data with different architectures (topologies and activation functions) in terms of the coefficient of correlation (r), the coefficient of determination (R^2^), the mean absolute error (MAE), and the root mean squared error (RMSE) for the training, test, and validation sets are shown in Table 5 and Figure 5. Table S1 (Supplementary Materials) reports the best five ANN architectures for each parameter.

Regarding TSS, high correlation was found with 17 neurons in the hidden layer, a logistic activation function for the hidden neurons and for the output neurons. The high values of r and R^2^ (0.925 and 0.855, respectively) and the low values of RMSE and MAE (1.319 and 0.080, respectively) for the test set demonstrated good predictive accuracy. Similarly, the best model for DM, carried out with 27 neurons in the hidden layer, an exponential activation function for the hidden neurons, and a hyperbolic tangent function for the output neurons, showed good predictive performance (r = 0.926; R^2^ = 0.857; RMSE = 1.379, MAE = 0.047 for the test set). Lastly, the model for TA, obtained with 17 neurons in the hidden layer and a logistic activation function for the hidden and the output neurons, presented unsatisfactory predictive ability due to the values of r and R^2^ being equal to 0.825 and 0.681, respectively, and the high values of the other metrics for the test set.

### 3.4. ANN Models Based on HSI Data

**Figure 6:**
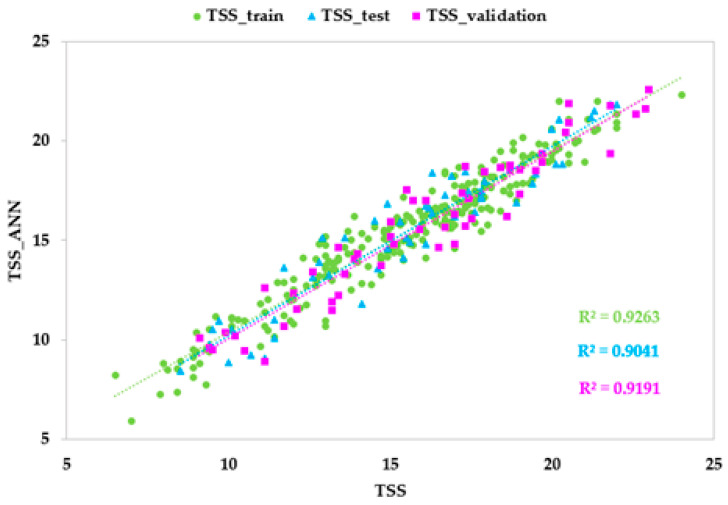

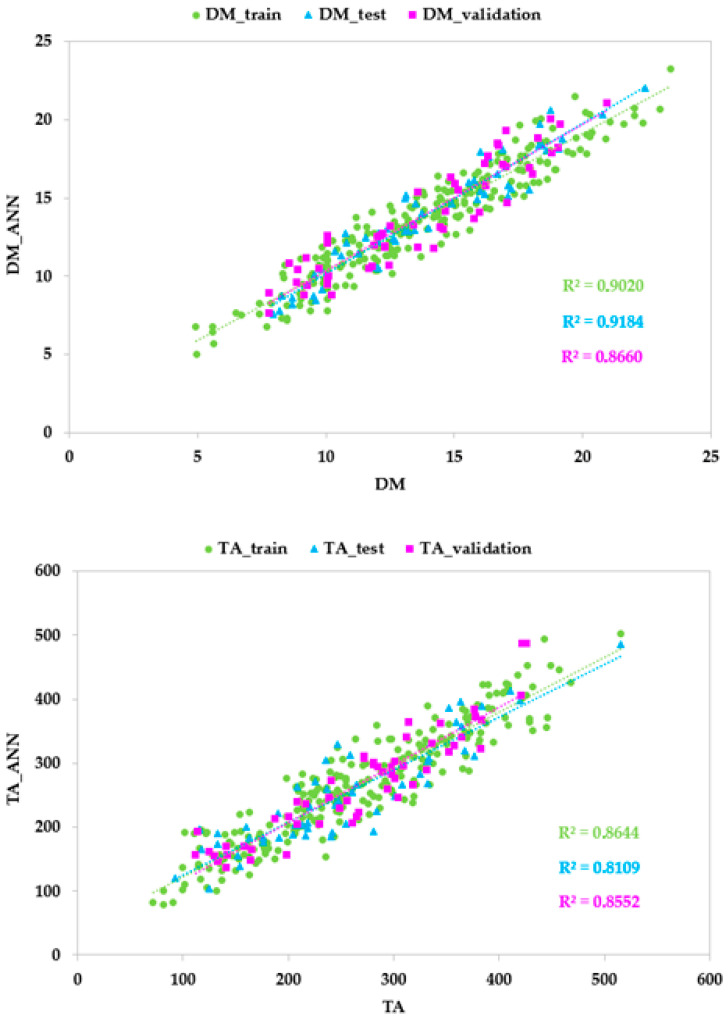
Predicted vs. experimental values of total soluble solids content (TSS), titratable acidity (TA), and dry matter (DM) using the optimal ANN topologies and HSI spectra. The coefficients of determination (R^2^) for the training, test, and validation sets are reported.

The predictive performances of the ANN models based on HSI data with different architectures (topologies and activation functions) in terms of the coefficient of correlation (r), the coefficient of determination (R^2^), the mean absolute error (MAE) and the root mean squared error (RMSE) for the training, test, and validation sets are shown in Table 6 and Figure 6. Table S2 (Supplementary Materials) reports the five best ANN architectures for each parameter.

The best model for TSS was developed with 31 neurons in the hidden layer, and an identity logistic activation function for the hidden and output neurons. The metrics for this model highlighted an optimal goodness of fit, with r and R^2^ values higher than 0.90, and low values of RMSE and MAE for all sets. The good performance also resulted in the model developed for DM, obtained with 20 neurons in the hidden layer, and an identity activation function for the hidden and output neurons. The high values of r and R^2^ (0.958 and 0.918, respectively), and the low values of RMSE and MAE (1.039 and 0.009, respectively) for the test set demonstrated high predictive accuracy. The optimal model for TA was developed with 50 neurons in the hidden layer, and a logistic activation function for the hidden and output neurons. The predictive ability result was good, with values of r and R^2^ equal to 0.901 and 0.811, respectively, and values of RMSE and MAE equal to 38.974 and 2.237, respectively, for the test set.

### 3.5. Sensitivity Analysis

To determine which input spectral variables had a greater impact on the output parameters, a sensitivity analysis was carried out for each ANN model (Table 7), in accordance with Pizarroso et al. [35] and Amoriello et al. [24]. If the independent variable is important, the global sensitivity should be large (>>1). The spectral regions, shown for both devices, corresponding to the second overtone of O–H and N–H, to the third overtone of C–H, and to a combination band of O–H bonds mostly affected TSS, TA, and DM, in agreement with Camps e Christen [6] and Pu et al. [34].

### 3.6. Validation of Developed Models with External Datasets

**Figure 7:**
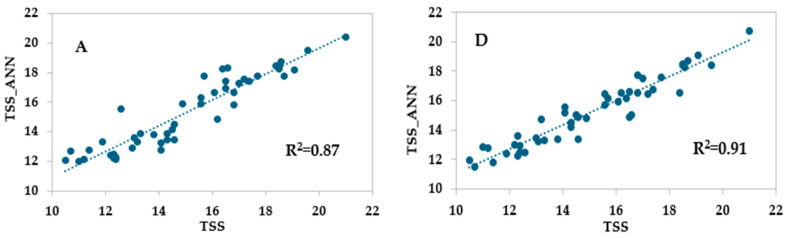

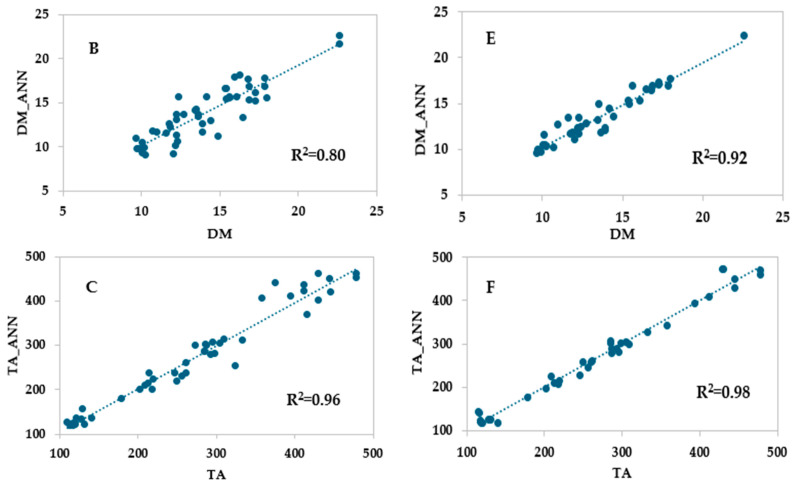
Predicted vs. experimental values of total soluble solid content (TSS), titratable acidity (TA), and dry matter (DM) using external Vis/NIR spectrophotometer dataset (**A**–**C**), external HSI dataset (**D**–**F**) and the optimal ANN topologies. The coefficients of determination (R^2^) are reported.

External validation is the evaluation of model performance in datasets that differ from those for model development. In our study, 48 quality and spectral data from four different cultivars, namely, Biotipo A, Ottavianese, Pellecchiella, and Perla, collected during the harvests of 2023 and 2024, were used to understand the generalizability of our results. The optimal topologies for each model and for the two devices were applied to the two external validation datasets. Figure 7 shows the deviations between the measured and predicted parameters and the coefficients of determination (R^2^). Overall, the performances obtained for the models built with HSI data were better than those with Vis/NIR data, in agreement with those obtained for the internal datasets. Moreover, an accuracy higher than 90% of the external validation means that the model is robust [36]. Following this criterion, it is possible to assert that the models obtained for the HSI dataset are robust, while a lower generalizability was found for the TSS and DM models of the Vis/NIR dataset.

## 4. Discussion

The evaluation of fruit quality is a crucial point for growers, producers, shippers, packers, retailers, and consumers. The presence of immature, overripe, or low-quality fruits on the market can negatively influence apricot consumption and reduce profits. Apricot quality is determined by many pre-harvest factors (genetics, environmental conditions, agronomic management systems), as well as post-harvest factors (textural problems, handling) [37].

In this work, the influence of the varieties and the annual environmental conditions on quality was considered. To do this, fruits of seventeen early-to-late cultivars representative of apricot species from a single orchard with the same agronomic management system were analyzed. Sample quality was determined by external traits and physico-chemical characteristics (Table 2, Table 3 and Table 4). In particular, the internal characteristics are considered the main descriptors of consumer acceptability, of the reiteration of the fruit purchase [38], and of the optimal harvest time [39]. In previous studies, Crisosto et al. [40] and Ceccarelli et al. [41] highlighted the relationship between the soluble solids content and the titratable acidity to evaluate consumers’ acceptability of fruits.

The results represented the expected variation in quality parameters, highlighting differences among cultivars (Table 4). Flesh firmness is considered a good index of maturity, and it is strongly dependent on genetic characteristics [42]. In our study, early cultivars showed higher FF values. Moreover, FF can also be used as a measure of eating quality because fruits with high firmness can cause difficulties in chewing [7]. The significant differences in terms of sugar content can be due to both specific genetic characteristics and to the different solar radiation during the May–July period, corresponding to the periods of early, intermediate, and late maturation. Solovchenko et al. [43] and Intrigliolo et al. [44] reported a significant relationship between the primary and secondary metabolism of plants and solar radiation. In fact, the biosynthesis of carbohydrates partially accumulated in fruits increases as the number of hours of sunshine per day increases. At the same time, as the number of hours of sunshine and temperatures increase, the plant is naturally exposed to water stress, which leads to a reduction in water in the fruit and, therefore, to an increase in total soluble solids. Many studies highlighted high variability in flesh color, flowering time, and soluble solids content in different apricot genotypes [37,42,45,46]. Regarding titratable acidity, higher values were detected for the early cultivars compared to the intermediate and late ones. This is mainly due to the genetic differences between the cultivars and, consequently, the primary and secondary metabolism of the plants themselves, as reported by Pennone et al. [47]. Similar results on different commercial apricot cultivars from large-scale retail trade were also reported in a previous study by Amoriello et al. [7]. Dry matter is a key factor used to assess both fruit’s susceptibility to handling and transportation and suitability for dehydration and processing of apricots into jams, jellies, and juices [48]. Generally, cultivars with low DM values are meant to fresh consumption, whereas drying and processing are preferred for cultivars with high DM values [48,49]. All cultivars showed DM values similar to those reported by Milosevic et al. [50], Drogoudi et al. [51], and Ruiz and Egea [52].

The spectral information in the Vis/NIR regions enabled the differentiation of the cultivars, capturing differences in the pigment content and macro components of the apricot samples. In fact, each pigment has a specific absorption spectrum in the visible region (300–750 nm), whereas the NIR region is related to the measurement of overtones and combination tones of molecular vibrations. For instance, carotenes and xanthophylls absorb at 464 and 435 nm, respectively; anthocyanins at 530–550 nm; sugar–protein complexes at 580 nm; and chlorophyll at around 680 nm [7,30,31,32]. High signal intensity in these spectral regions could be correlated to the samples with high values of b* and a* (Table 2), indicating a very light or not red blush color, as reported by Sartori et al. [20] and Della Strada et al. [19]. Moreover, the NIR region is characterized by the overtones of the OH groups related to water (780–970 nm), the second overtone of the O–H and N–H groups, the combination bands of the O–H bonds, and of the third overtone of the C– bonds (at around 830 nm, and between 950 and 1000 nm) [5,6,33,34]. Indeed, this spectral region is closely related to the carbohydrate, sugar, and water absorbance bands, which showed the maximum intensity for Portici and Cafona I, correspondent to high DM and TSS contents.

The second aim of this study was to verify the ability of portable Vis/NIR instruments (a Vis/NIR spectrophotometer and a hyperspectral imaging device) to provide measurements of apricots’ internal quality attributes in real time in a non-invasive and non-destructive manner, and requiring minimal sample preparation. The use of these spectrum-based instruments in fruit quality monitoring is growing enormously, also due to the possibility of the simultaneous assessment of multiple parameters, with the purpose of replacing traditional analytical methods [53]. Although several studies have been conducted on the non-destructive quality assessment of apricots, especially for Vis/NIR spectrophotometers, some aspects still need to be explored. Among these is the need to obtain robust calibration models, and therefore an appropriate choice of prediction algorithms, which are able to take into account all genetic, agronomic, and environmental factors [54]. In previous studies, model calibrations for determining fruit quality attributes were obtained using multiple linear regression or partial least squares (PLS) algorithms, thus assuming linear relationships between input and output variables [4,5,6,8]. In recent years, the spread of artificial intelligence techniques has made prediction models more efficient. In particular, artificial neural networks (ANNs) have been widely used with the advantage of not making any assumptions about the data distribution or the relationship between quality attributes and spectral variables [11,14,15,16,17]. In our study, we successfully applied artificial neural networks to predict total soluble solid content and dry matter, and, to a lesser extent, titratable acidity. Differently from previous studies, these models were built with samples from many apricot cultivars and harvested in two years, effectively making the models multi-cultivar and multi-year in nature. External validation was crucial for establishing machine learning model quality and for determining model efficacy and generalizability. In fact, internal validation techniques, such as cross-validation and bootstrap, cannot guarantee the accuracy or the robust sensitivity and specificity of machine learning models due to potentially biased training data and the complexity of the validation procedure itself [36]. Although the internal validation approach, which consists of splitting one input dataset into parts (a training subset, a test subset, and a validation subset) is more economic, validation with an external dataset can avoid yielding overly optimistic performance estimates. On the other hand, an additional external dataset entails an additional cost in terms of time and money, which are not always available. This is often the reason why few studies make use of external validation. Another element to consider is the finding of the optimal external sample sizes. Also in this case, economic and temporal availability is crucial. Our results are encouraging for the prediction of the internal quality of apricot fruit, especially if the HSI device is used. However, taking into account the different cost of the two instruments, the end user must evaluate whether the lower predictive ability of the Vis/NIR spectrophotometer is acceptable in the face of a significantly lower cost of the instrument and ease of use.

Furthermore, the biggest challenge is to create low-cost portable instruments, making these technologies more widely applicable. To do this, it is necessary to identify the spectral regions that affect the models the most. In fact, the selection of most predictive spectral bands can (i) improve the prediction ability by eliminating uninformative or interfering variables; (ii) reduce the risk of overfitting; (iii) increase the computational speed of prediction by reducing the number of possible combinations; and (iv) allow the building of instruments for the accurate prediction of the internal qualitative attributes on low spectral ranges, providing a more stable result and allowing considerable savings in the production costs of the instrument [55]. The sensitivity analysis allowed us to select wavelengths from the regions corresponding to the second overtone of O–H and N–H, to the third overtone of C–H, and to a combination band of O–H bonds, proving to be the most informative and predictive bands for the TSS, TA, and DM models. These spectral regions have been confirmed to be related to TSS, TA, and DM in previous studies [6,34].

## 5. Conclusions

In this work, the potential of a portable Vis/NIR spectrophotometer (310–1100 nm) and of a portable hyperspectral imaging device (400–1000 nm) for evaluating the quality of apricot fruit in a sustainable, non-destructive, and rapid way was investigated. Spectral information allowed cultivars to be discriminated, highlighting the spectral regions that most influenced the clustering of the seventeen cultivars. Cultivar discrimination can help breeders, producers, etc., to enhance the product’s marketability, according to quality and nutritional attributes. Furthermore, cultivar-independent and multi-year models based on spectral data and artificial neural networks were built for three physico-chemical attributes (total soluble solids content, titratable acidity, and dry matter). High accuracy and predictive power were obtained, especially for total soluble solids content and dry matter, whereas lower prediction accuracy was found for titratable acidity. Moreover, the most predictive spectral regions were identified for all qualitative parameters. These results can favor the development of rapid, non-destructive, and low-cost portable tools for monitoring fruit quality, enhancing the efficiency of the apricot supply chain. It could be necessary further studies to use these systems for the real-time prediction and classification of apricots in a sorting line, refining the models and testing them on additional cultivars.

## Figures and Tables

**Table 1:** Geographical origin, ripening time, and parental lines of genotypes analyzed, according to the varietal lists [[19,20], https://plantgest.imagelinenetwork.com (accessed on 10 November 2024)]. * Number of days before ripening of Goldrich (18 June).

Genotype	Pedigree	Origin	Ripening Time *
Aurora	*Goldrich* × *Ouardi*	Italy (Emilia Romagna)	Early (−21)
Bella d’Imola	*Open pollination*	Italy (Emilia Romagna)	Early-Medium (−7)
Bora	*Early Blush × PA7005-2 (Rival × PA 63-265)*	Italy (Emilia Romagna)	Early-Medium (−14)
Bulida	*Unknown*	Spain	Medium-Late (9)
Cafona I	*Unknown*	Italy (Campania)	Medium (0)
Krupna Skopjanka	*Unknown*	Macedonia	Medium (0)
Marietta Milady	*S. Francesco* × *Polonais*	Italy (Tuscany)	Medium (6)
Muscat	*Unknown*	France	Medium-Late (9)
Nella	*Unknown*	Italy (Campania)	Early (−21)
Ninfa	*Ouardi* × *Thyrinthos*	Italia (Emilia Romagna)	Early (−21)
NJA42	*Unknown*	Italy (Tuscany)	Medium-Late (10)
Portici	*Unknown*	Italy (Campania)	Medium-Late (9)
Precoce Toscana	*Unknown*	Italy (Tuscany)	Medium (0)
Rubista	*Unknown*	Unknown	Early-Medium (−14)
S. Castrense	*Unknown*	Italy (Campania)	Medium (6)
Tardiva di Bordaneil	*Unknown*	France	Early (−18)
Thyrinthos	*Unknown*	Greece	Early-Medium (−14)

**Table 2:** Pomological characteristics (mean ± standard deviations) of 17 apricot genotypes. Differences between letters in the same column indicate significant differences (*p* < 0.05).

CV	Weight (g)	Longitudinal Diameter (mm)	Lateral Diameter (mm)	Equatorial Diameter (mm)
Aurora	45 ± 23 bcd	40 ± 3 cd	38 ± 4 cd	40 ± 2 cd
Bella di Imola	69 ± 12 a	51 ± 4 a	49 ± 5 a	47 ± 2 a
Bora	70 ± 11 a	50 ± 4 a	48 ± 3 a	47 ± 3 a
Bulida	59 ± 11 b	47 ± 4 b	48 ± 4 a	46 ± 4 ab
Cafona I	39 ± 5 d	38 ± 3 d	39 ± 3 cd	38 ± 3 d
Krupna Skopjanka	46 ± 8 c	42 ± 3 c	45 ± 3 ab	40 ± 2 cd
Marietta Milady	54 ± 6 b	44 ± 4 b	43 ± 3 bc	44 ± 2 b
Muscat	30 ± 4 e	38 ± 2 d	36 ± 3 d	34 ± 3 e
Nella	56 ± 7 b	44 ± 2 bc	46 ± 2 ab	46 ± 3 ab
Ninfa	43 ± 10 cd	41 ± 3 cd	41 ± 4 bc	41 ± 3 c
NJA42	57 ± 15 b	48 ± 6 ab	43 ± 4 bc	44 ± 4 b
Portici	50 ± 9 bc	48 ± 9 ab	42 ± 5 bc	41 ± 2 c
Precoce Toscana	47 ± 4 c	46 ± 4 b	43 ± 3 bc	39 ± 2 d
Rubista	53 ± 11 b	42 ± 4 c	43 ± 3 bc	41 ± 3 c
S. Castrense	39 ± 5 d	39 ± 2 d	39 ± 3 cd	38 ± 2 d
Tardiva di Bordaneil	66 ± 10 ab	47 ± 3 b	48 ± 3 a	48 ± 2 a
Thyrinthos	51 ± 8 bc	45 ± 4 b	44 ± 2 b	43 ± 2 bc

**Table 3:** Mean values ± standard deviations of CIELab coordinates (L*, a*, b*) for each cultivar. Differences between letters in the same column indicate significant differences (*p* < 0.05).

CV	L*	a*	b*
Aurora	55.99 ± 6.54 a	12.82 ± 6.18 bcde	36.38 ± 6.63 cd
Bella di Imola	59.87 ± 4.11 a	18.75 ± 3.88 b	44.24 ± 4.39 a
Bora	57.70 ± 4.12 a	19.39 ± 4.18 b	41.08 ± 5.12 ab
Bulida	59.44 ± 4.93 a	11.28 ± 3.31 de	43.88 ± 6.28 a
Cafona I	59.70 ± 4.77 a	10.88 ± 3.96 e	33.07 ± 6.25 d
Krupna Skopjanka	59.13 ± 4.88 a	14.60 ± 2.97 cd	40.92 ± 6.20 ab
Marietta Milady	60.67 ± 4.66 a	14.80 ± 2.40 cd	41.14 ± 7.09 ab
Muscat	57.30 ± 6.77 a	14.24 ± 3.11 cd	34.40 ± 7.23 d
Nella	57.49 ± 5.60 a	15.39 ± 5.79 c	40.81 ± 6.34 ab
Ninfa	61.78 ± 6.35 a	12.15 ± 4.02 cde	39.13 ± 7.50 bc
NJA42	60.80 ± 4.21 a	14.67 ± 2.47 cd	42.85 ± 3.92 ab
Portici	61.50 ± 7.90 a	12.76 ± 3.76 bcde	43.10 ± 9.60 ab
Precoce Toscana	58.89 ± 4.04 a	13.84 ± 4.12 bcd	44.44 ± 3.51 a
Rubista	36.42 ± 4.49 b	29.25 ± 5.33 a	13.65 ± 4.99 e
S. Castrense	56.99 ± 4.21 a	15.35 ± 2.66 c	32.97 ± 6.60 d
Tardiva di Bordaneil	62.30 ± 9.07 a	19.12 ± 5.99 b	41.70 ± 6.39 ab
Thyrinthos	59.81 ± 9.08 a	18.86 ± 8.71 b	41.85 ± 9.48 ab

**Table 4:** Mean values ± standard deviations of quality traits of 17 apricot genotypes. FF (N) = firmness; TSS (g 100 g^−1^ FW) = total soluble solids content; TA (mEq 100 L^−1^) = titratable acidity; DM (g 100 g^−1^ FW) = dry matter; FW = fresh weight. Differences between letters in the same column indicate significant differences (*p* < 0.05).

CV	FF (N)	TSS (g 100 g^−1^ FW)	DM (g 100 g^−1^ FW)	TA (mEq 100 L^−1^)
Aurora	10 ± 4 ef	14.9 ± 2.3 cd	14.2 ± 1.9 d	391 ± 61 a
Bella di Imola	46 ± 29 a	15.5 ± 2.6 c	12.4 ± 1.8 e	271 ± 37 d
Bora	47 ± 36 a	14.1 ± 3.0 d	12.0 ± 2.7 ef	358 ± 50 b
Bulida	13 ± 7 ef	17.3 ± 1.0 b	17.5 ± 1.7 b	203 ± 79 e
Cafona I	10 ± 8 f	19.2 ± 1.6 a	17.7 ± 1.6 b	154 ± 22 fg
Krupna Skopjanka	8 ± 4 f	17.3 ± 0.7 b	15.4 ± 1.7 c	320 ± 30 c
Marietta Milady	31 ± 28 bc	15.2 ± 1.6 cd	12.4 ± 1.9 e	264 ± 37 d
Muscat	11 ± 8 ef	19.6 ± 2.1 a	19.3 ± 2.5 a	173 ± 27 f
Nella	17 ± 9 def	12.3 ± 2.7 e	10.5 ± 2.4 gh	260 ± 26 d
Ninfa	22 ± 9 cde	11.4 ± 3.3 e	9.4 ± 1.9 i	206 ± 76 e
NJA42	38 ± 34 ab	15.1 ± 1.2 cd	12.0 ± 1.4 ef	202 ± 59 e
Portici	19 ± 15 def	19.5 ± 1.8 a	17.0 ± 2.5 b	130 ± 30 g
Precoce Toscana	15 ± 9 ef	15.1 ± 1.7 cd	12.1 ± 0.9 ef	272 ± 53 d
Rubista	14 ± 15 ef	15.9 ± 1.3 c	12.9 ± 1.8 e	417 ± 43 a
S. Castrense	19 ± 9 def	19.0 ± 1.9 a	15.9 ± 2.2 c	275 ± 53 d
Tardiva di Bordaneil	26 ± 13 cd	12.4 ± 2.6 e	11.0 ± 1.9 fg	285 ± 64 d
Thyrinthos	26 ± 7 cd	11.2 ± 2.1 e	9.8 ± 1.6 hi	315 ± 30 c

**Table 5:** Neural network architectures, regression metrics for the highest training, test, and validation set predictions, goodness of fit, and residual analysis for the developed ANN models based on Vis/NIR data. MLP = multilayer perceptron; Tanh = hyperbolic tangent function; Exp = exponential function; TSS = total soluble solids content (g 100 g^−1^ FW); TA = titratable acidity (mEq L^−1^); DM = dry matter (g 100 g^−1^ FW); r = coefficient of correlation; R^2^ = coefficient of determination; MAE = mean absolute error; RMSE = root mean squared error.

		TSS	DM	TA
	Topology (input–hidden–output)	MLP (260–17–1)	MLP (260–27–1)	MLP (260–17–1)
Activation Function	Hidden Neurons Output Neurons	Logistic Logistic	Exp Tahn	Logistic Logistic
Training set	r	0.947	0.898	0.819
	R^2^	0.898	0.806	0.671
	RMSE	1.048	1.535	51.814
	MAE	0.049	0.045	1.110
Test set	r	0.925	0.926	0.825
	R^2^	0.855	0.857	0.681
	RMSE	1.319	1.379	55.073
	MAE	0.080	0.047	0.376
Validation set	r	0.939	0.929	0.810
	R^2^	0.883	0.863	0.656
	RMSE	1.286	1.310	53.588
	MAE	0.031	0.032	2.374

**Table 6:** Neural network architectures, regression metrics for the highest training, test, and validation sets predictions, goodness of fit, and residual analysis for the developed ANN models based on HSI data. MLP = multilayer perceptron; Tanh = hyperbolic tangent function; Exp = exponential function; TSS = total soluble solids content (g 100 g^−1^ FW); TA = titratable acidity (mEq L^−1^); DM = dry matter (g 100 g^−1^ FW); r = coefficient of correlation; R^2^ = coefficient of determination; MAE = mean absolute error; RMSE = root mean squared error.

		TSS	DM	TA
	Topology (input–hidden–output)	MLP (260–31–1)	MLP (260–20–1)	MLP (260–50–1)
Activation Function	Hidden Neurons Output Neurons	Identity Identity	Identity Identity	Logistic Logistic
Training set	r	0.962	0.950	0.930
	R^2^	0.926	0.902	0.864
	RMSE	0.935	1.187	34.32
	MAE	0.084	0.159	0.655
Test set	r	0.951	0.958	0.901
	R^2^	0.904	0.918	0.811
	RMSE	1.103	1.039	38.974
	MAE	0.055	0.009	2.237
Validation set	r	0.959	0.931	0.925
	R^2^	0.919	0.866	0.855
	RMSE	1.112	1.288	32.741
	MAE	0.300	0.090	0.953

**Table 7:** Main sensitive spectral ranges from sensitivity analysis for each ANN model; TSS = total soluble solids content; TA = titratable acidity; DM = dry matter.

	TSS	DM	TA
Vis/NIR spectrophotometer (310–1100 nm)	410–420 740–760 850–920 950–970	380–420 470–490 740–770 840–1000	320–350 410–500 530–550 655–665 720–920 990–1060
HSI device (400–1000 nm)	460–480 730–790 800–920 940–990	400–440 730–780 850–890 900–980	400–430 480–520 540–570 630–700 730–770 870–950

## Data Availability

The original contributions presented in the study are included in the article/Supplementary Materials, further inquiries can be directed to the corresponding author.

## Supplementary Materials

The following supporting information can be downloaded at: https://www.mdpi.com/article/10.3390/foods14020196/s1, Table S1: Neural network architectures and correlation coefficients for the developed ANN models based on Vis/NIR data. The best architecture for each parameter was highlighted in bold; Table S2: Neural network architectures and correlation coefficients for the developed ANN models based on hyperspectral data. The best architecture for each parameter was highlighted in bold.
