# Supplementary material for: Vis/NIR Spectroscopy and Vis/NIR Hyperspectral Imaging for Non-Destructive Monitoring of Apricot Fruit Internal Quality with Machine Learning
Source: Foods. 2025 Jan 10;14(2):196. doi: 10.3390/foods14020196 (PMC11764486; doi:10.3390/foods14020196)
Supplement: Supplementary file 1 [file foods-14-00196-s001.zip › foods-3355078-supplementary.pdf]

Vis/NIR spectroscopy and Vis/NIR hyperspectral imaging for non-destructive monitoring of apricot fruit internal quality with machine learning

Tiziana Amoriello[1,*], Roberto Ciorba[2], Gaia Ruggiero[2], Francesca Masciola[2], Daniela Scutaru[2], Roberto Ciccoritti[2,*]

Supplementary materials

Supplementary Table S1. Neural network architectures and correlation coefficients for the developed ANN models based on Vis/NIR data. The best architecture for each parameter was highlighted in bold.

	Neurons in hidden layers	Activation Function		Training Set	Test Set	Validation set
		Hidden Neurons	Output Neurons	Correlation coefficient	Correlation coefficient	Correlation coefficient
TSS	**17**	**Logistic**	**Logistic**	**0.947**	**0.925**	**0.939**
	22	Logistic	Identity	0.935	0.922	0.937
	43	Exp	Exp	0.933	0.919	0.934
	37	Tanh	Logistic	0.933	0.911	0.932
	28	Tanh	Identity	0.934	0.923	0.931
DM	**27**	**Exp**	**Tanh**	**0.898**	**0.926**	**0.929**
	30	Logistic	Identity	0.907	0.908	0.925
	22	Logistic	Tanh	0.886	0.924	0.920
	12	Exp	Tanh	0.891	0.918	0.918
	35	Identity	Exp	0.903	0.905	0.924
TA	9	Exp	Logistic	0.833	0.819	0.766
	**17**	**Logistic**	**Logistic**	**0.819**	**0.825**	**0.810**
	28	Logistic	Logistic	0.910	0.804	0.789
	38	Tanh	Exp	0.822	0.824	0.793
	25	Logistic	Exp	0.825	0.807	0.788

Legend: MLP = multilayer perceptron; Tanh = hyperbolic tangent function; Exp = exponential function; TSS = total soluble solid content; TA = titratable acidity; DM = dry matter.

Supplementary Table S2. Neural network architectures and correlation coefficients for the developed ANN models based on hyperspectral data. The best architecture for each parameter was highlighted in bold.

	Neurons in hidden layers	Activation Function		Training Set	Test Set	Validation set
		Hidden Neurons	Output Neurons	Correlation coefficient	Correlation coefficient	Correlation coefficient
TSS	45	Identity	Logistic	0.964	0.897	0.910
	11	Identity	Tanh	0.956	0.889	0.887
	**31**	**Identity**	**Identity**	**0.962**	**0.951**	**0.959**
	16	Identity	Identity	0.946	0.934	0.934
	39	Identity	Logistic	0.947	0.900	0.913
DM	32	Identity	Identity	0.902	0.924	0.925
	47	Identity	Logistic	0.938	0.910	0.928
	36	Identity	Tanh	0.892	0.922	0.907
	**20**	**Identity**	**Identity**	**0.950**	**0.958**	**0.931**
	15	Exp	Logistic	0.923	0.938	0.923
TA	**50**	**Logistic**	**Logistic**	**0.930**	**0.901**	**0.925**
	25	Identity	Tanh	0.897	0.895	0.899
	36	Logistic	Tanh	0.837	0.849	0.898
	50	Exp	Identity	0.890	0.886	0.902
	45	Logistic	Logistic	0.920	0.876	0.896

Legend: MLP = multilayer perceptron; Tanh = hyperbolic tangent function; Exp = exponential function; TSS = total soluble solid content; TA = titratable acidity; DM = dry matter.
